# Sense and Antisense *DMPK* RNA Foci Accumulate in DM1 Tissues during Development

**DOI:** 10.1371/journal.pone.0137620

**Published:** 2015-09-04

**Authors:** Lise Michel, Aline Huguet-Lachon, Geneviève Gourdon

**Affiliations:** 1 Inserm UMR 1163, Paris, France; 2 Paris Descartes—Sorbonne Paris Cité University, Imagine Institute, Paris, France; University of Valencia, SPAIN

## Abstract

Myotonic dystrophy type 1 (DM1) is caused by an unstable expanded CTG repeat located within the *DMPK* gene 3’UTR. The nature, severity and age at onset of DM1 symptoms are very variable in patients. Different forms of the disease are described, among which the congenital form (CDM) is the most severe. Molecular mechanisms of DM1 are well characterized for the adult form and involve accumulation of mutant *DMPK* RNA forming foci in the nucleus. These RNA foci sequester proteins from the MBNL family and deregulate CELF proteins. These proteins are involved in many cellular mechanisms such as alternative splicing, transcriptional, translational and post-translational regulation miRNA regulation as well as mRNA polyadenylation and localization. All these mechanisms can be impaired in DM1 because of the deregulation of CELF and MBNL functions. The mechanisms involved in CDM are not clearly described. In order to get insight into the mechanisms underlying CDM, we investigated if expanded RNA nuclear foci, one of the molecular hallmarks of DM1, could be detected in human DM1 fetal tissues, as well as in embryonic and neonatal tissues from transgenic mice carrying the human *DMPK* gene with an expanded CTG repeat. We observed very abundant RNA foci formed by sense *DMPK* RNA and, to a lesser extent, antisense *DMPK* RNA foci. Sense *DMPK* RNA foci clearly co-localized with MBNL1 and MBNL2 proteins. In addition, we studied *DMPK* sense and antisense expression during development in the transgenic mice. We found that *DMPK* sense and antisense transcripts are expressed from embryonic and fetal stages in heart, muscle and brain and are regulated during development. These results suggest that mechanisms underlying DM1 and CDM involved common players including toxic expanded RNA forming numerous nuclear foci at early stages during development.

## Introduction

Myotonic dystrophy type 1 (DM1) is a dominantly inherited neuromuscular disorder characterized by very variable symptoms. Several forms of DM1 can be differentiated: a late onset form with pre-senile cataracts and early frontal balding; an adult form which presents myotonia, insulin resistance, cardio-respiratory problems, muscle weakness, hypersomnia and cognitive impairments; and the congenital form (CDM), the most severe form. CDM is characterized by high perinatal mortality (30–40%), mainly due to respiratory distress, hypotonia, sucking and swallowing difficulties. Children who survive the neonatal period will later show delays in motor development and intellectual disability [1, 2]. Interestingly, no evident congenital form is observed in DM2, another form of myotonic dystrophy showing similar symptoms in adult, although one case has been described [3].

The genetic mutation causing DM1 and CDM has been identified as the expansion of an unstable CTG repeat, in the 3’ untranslated region (3’UTR) of a gene encoding a protein kinase (*DMPK*) [4–7]. The normal *DMPK* gene contains 5–37 CTG repeats in the 3'UTR, while all DM1 patients have repeats expanding from 50 to several thousand CTG trinucleotides in CDM. The size of the CTG repeat, which increases from generation to generation, is generally correlated with clinical severity and lower age at onset, providing a molecular basis for anticipation observed in DM1 families [1]. Clinically similar to DM1, DM2 is caused by the expansion of an intronic CCTG repeat in an unrelated gene [8]. The identification of the genetic defect underlying DM2 and the development of transgenic mice [9] emphasized the major role of an RNA *trans-*dominant effect in myotonic dystrophy pathogenesis. CUG (or CCUG) repeat-containing RNA aggregates in the nucleus forming nuclear RNA foci [10, 11]. These nuclear foci sequester RNA-binding proteins such as the muscleblind-like splicing regulator (MBNL) family, affecting their function in the nucleus [12]. In addition, CUG-BP1 (or CELF1, a member of the CELF family) is up-regulated in DM1 heart and skeletal muscle through a PKC-mediated phosphorylation event, which stabilizes the protein [13, 14]. Activity of ETR3, or CELF2 another member of the CELF family, is also affected in DM1 [15]. MBNL and CELF proteins antagonistically regulate alternative splicing transitions during normal development [16]. In addition, MBNL and CELF proteins have many functions besides splicing regulation [17–20]. Depletion of MBNL by CUG repeats and increased expression of CUGBP1 result in transcriptional and posttranscriptional defects, including alterations in splicing, polyadenylation, mRNA stability, localization, splicing and miRNA deregulation [12] [21].

An antisense transcript emanating from the adjacent *SIX5* regulatory region downstream of the CTG repeat has been described [22]. This transcript starts downstream of the CTG repeat that is surrounded by CTCF binding sites. CTCF binds to sites flanking the CTG repeats at the non-expanded *DM1* locus, to form a chromatin insulator element. In CDM myoblasts, the expanded allele is associated with loss of CTCF binding and propagation of heterochromatin [22, 23]. It has been proposed that loss of insulator function due to impaired CTCF binding on the expanded allele in CDM might also result in higher expression levels of mutant *DMPK* late in embryogenesis. Expression of high levels of expanded CUG repeat-containing RNA at this stage might contribute to the earlier disease phenotype in CDM. More recently, it has been reported that RNA carrying CTG/CAG expansions can be translated by repeat associated non-ATG (RAN) translation in homopolymeric proteins in the absence of an ATG start codon. Homopolymeric proteins generated by antisense *DMPK* mutant RNA can form potentially toxic aggregates found in DM1 mouse models and human tissues [24]. The contribution of antisense RNA to DM1 pathology remains unknown but it adds another level of complexity to the possible mechanisms involved in this disease. Furthermore, the relationship between the antisense RNA, the expression levels of *DMPK* and the CTG repeat length is still unclear during development.

Until now, the mechanisms of DM1 were mainly studied in the context of the adult form and few models have recreated early features of DM1. Furthermore, the extent and distribution of RNA foci during development is unknown. An inducible mouse model overexpressing the *DMPK* 3’UTR from embryonic stages reproduced cardinal features of DM1 from 2 weeks of age, more severely than in adult mice [25]. A zebrafish model injected with (CUG)91 containing RNA in single-cell embryo showed toxicity in the nervous system and in muscle during development [26]. In this study, we used human fetal samples as well as mouse models that we previously developed by the insertion of a human 45 kb transgene expressing the *DMPK* gene with normal (DM20) or expanded (DMSXL) CTG repeats under the control of its own human promoter [27, 28]. We investigated the extent of RNA foci accumulation throughout different developmental stages. We found very abundant foci in human and mouse DM1 embryos and fetuses and observed that sense and antisense *DMPK* RNA are both expressed from early stages and are developmentally regulated.

## Materials and Methods

### Animals

DMSXL and DM20 mice (>90% C57BL/6 background) carried 45 kb of human genomic DNA cloned from normal or mutated DM1 patient allele as previously described [27]. Transgenic status was assayed by PCR [29]. Housing and handling of mice were performed in accordance with the guidelines established by the French Council on animal care “Guide for the Care and Use of Laboratory Animals”: EEC86/609 Council Directive—Decree 2001–131. The DMSXL mice used in this study derived from the DM300, after large expansion events [28], and carried between 1000 and 1600 CTG repeats, with a mean of 1250+/−110 CTG. The DM20 mice carried normal *DMPK* allele with 20 CTG repeats. The animals were only used for tissues recovering after sacrifice. This project has been conducted with the authorization for animal experimentation n° 75 003 in the animal facility with the approval n°: B 91 228 107 both delivered by Prefecture de police and the French veterinary department.

### Human Samples

Post mortem tissues of DM1 infants and tissues of fetuses were sampled at autopsy at least 18 years ago and stored at-80°C. No law required approvals at the time of tissues collection. The diagnosed CTG repeats ranged between 1100 and 2500 CTG.

### RNA isolation

Tissues were homogenized in Trizol (Life Technologies) using a tissue lyser (Retsch MM400): twice for 2 min 30 sec at 23 pulses/sec with 2 stainless 5 mm steel beads (Qiagen). After chloroform extraction, the aqueous phase was mixed with 70% ethanol and transferred to a Spin Cartridge of the PureLink RNA Mini Kit (Ambion by Life Technologies). Total RNA was extracted according to the manufacturer's protocol. A PureLink DNase step was inserted in the protocol, after binding of the RNA to the column as recommended. The quantity of RNA was determined by absorbance at 260 nm with a Nanodrop spectrophotometer and its quality was verified on a 1% agarose gel. Each RNA was controlled for absence of DNA contamination by RT-PCR using for RT random primers and for PCR primers located in exons 3 and 4 of the human *DMPK* gene (HS3: 5'-TGAAGATGAAGCAGACGG-3'; HS4: 5'-TCCCCATTCACCAACA-3') and in exons 4 and 5 of the mouse *Dmpk* gene (mdmCG1: 5'-GGAAGAAAGGGATGTATTA-3'; mdmCG2: 5'-CTCAGCAGCGTTAGCA-3'). Additional controls were performed using the same primers but without reverse transcriptase.

### Reverse transcription

For strand-specific RT-PCR, we attached a linker sequence, LK 5’-CGACTGGAG CACGAGGACACTGA-3’, to the 5’ end of a primer specific for the antisense or sense strands of *DMPK* using the strategy described by Cho et al [22]. cDNA was synthesized from 0,125 μg, 0,25 μg, 0,5 μg, 1 μg and 2 μg of RNA using Superscript III Reverse Transcriptase (Life Technologies). RNA, oligonucleotide primers (50 μmol) and dNTPs (10 μmol) were mixed and incubated at 65°C during 5 minutes. DTT (0,005 M final), First-Strand Buffer (1X final) and Superscript III RT (200 units) were added in final volume of 20 μl, and the mix was incubated 5 minutes at room temperature, then 1 hour at 55°C and 15 minutes at 70°C. The cDNA was treated with RNase A (0,05 μg/μl final) for 20 minutes at 37°C.

For each RT with different quantity of RNA input, we performed a quantitative RT-PCR (qRT-PCR). We analysed the results of qRT-PCR and compared with theoretical results obtained with cDNA from 0,125 μg, 0,25 μg, 0,5 μg, 1 μg and 2 μg of RNA. The RNA quantity was selected when the quantification of the studied gene was proportional to RNA quantity used for the RT. This step was made for each tissue and each gene studied. See Table 1 for oligonucleotide primer sequences.

**Table 1:** List of oligonucleotide primers, with linker sequence underlined, used for qRT-PCR experiments.

*DMPK* sense	Forward	CGACTGGAGCACGAGGACACTGA
	Reverse	GGAGAGGGACGTGTTG
	Oligo for RT	CGACTGGAGCACGAGGACACTGA CTTGCTCAGCAGTGTCA
*DMPK* antisense	Forward	GGAGCACGAGGACACTGA
	Reverse	TGCGAACCAACGATAG
	Oligo for RT	CGACTGGAGCACGAGGACACTGA CTTTCTTTCGGCCAGGCTGAGGC
*Dmpk* sense	Forward	CGACTGGAGCACGAGGACACTGA
	Reverse	GGAAGAAAGGGATGTATTA
	Oligo for RT	CGACTGGAGCACGAGGACACTGACTCAGCAGCGTTAGCA
18S	Forward	CAGTGAAACTGCGAATGG
	Reverse	CGGGTTGGTTTTGATCTG
	Oligo for RT	CGGGTTGGTTTTGATCTG

### Quantitative RT–PCR

The transcripts were amplified in a 7300 Real Time PCR System (Applied Biosystems) using Power SybrGreen detection (Life Technologies). Annealing temperatures and sample dilutions were optimized for each amplicon. For qRT-PCR, we used standard curves established with 6 known dilutions of plasmid carrying the amplicon. Samples were quantified in triplicate and experiments were repeated at least twice. *18S* rRNA was used as internal control. mRNA levels were quantified relative to *18S* transcripts and the ratios were normalized using the same standard sample. For experiments using mouse samples, RNAs from 4 to 6 sex- and age-matched animals were pooled. Oligonucleotide primer sequences are described in Table 1.

### FISH

Frozen sections of tissues (10 μm) were prepared: transversally for mouse embryo brains, and sagittally for muscle and heart studies. Sense ribonuclear inclusions were detected with a 5’-Cy3-labeled (CAG)_5_ PNA probe as described [30]. For antisense ribonuclear inclusion detection, we used a 5’-Cy3-labeled (CTG)_5_ PNA probe using the same protocol. For simultaneous FISH detection of sense and antisense foci, we first performed hybridization using the 5’-Alexa 488-(CAG)_5_ PNA probe to detected sense ribonuclear inclusions. After 3 washes in 1X PBS for 2 min at room temperature, the slides were hybridized with 5’-Cy3-labeled (CTG)_5_ PNA probe. The specificity of the RNA foci was determined by treating some slices before FISH detection with either RNase A (100 μg/mL in 2× SSC) or DNase (PureLink® DNase, 2 U/μL in DNase buffer) at 37°C for 30 min as shown in S2 Fig. Co-localization experiments with MBNL1 and MBNL2 were performed as previously described [30]. We used MBNL1 MB1a and MBNL2 MB2a antibodies, kindly provided by Ian Holt [31]. Images were acquired using a Confocal Leica TCS SP5 to quantify foci intensity (x100) or Zeiss Apotome to determine foci number and their co-localization (x63). The intensity quantification was performed on sum slice intensity of images using the ImageJ software (U. S. National Institutes of Health, Bethesda, Maryland, USA). The foci were delimited using the threshold of detection to define regions of interest (ROI). The ROI were filtered and excluded if it measured less than 4 pixels. By binary process, the ROI were dilated to match with real foci surface and the intensity was quantified. For each tissue (heart, muscle and brain), foci intensity was measured in 100–150 nuclei, throughout different regions of the tissue in 3 different embryos or neonates. The number of nuclei were counted using 3D reconstituted images with the Imaris software (BITPLANE). The mean intensity per nucleus was calculated for each sample and plotted. The number of foci, their nuclear localization and their co-localization with MBNL proteins were also checked in 3D reconstituted images, using the Imaris software (BITPLANE).

### Statistical analysis

All statistical analyses were performed with the GraphPad Prism software (GraphPad Software Inc.) using two-tailed Student's t-test when distributions were normal with equal or unequal variance as appropriate and Mann-Whitney test (for non-Gaussian distributions). The significance level was set at 0.05 for all statistical analyses.

## Results

### Expanded sense *DMPK* RNA accumulates in numerous nuclear foci at various embryonic, fetal and neonatal stages

**Fig 1:**
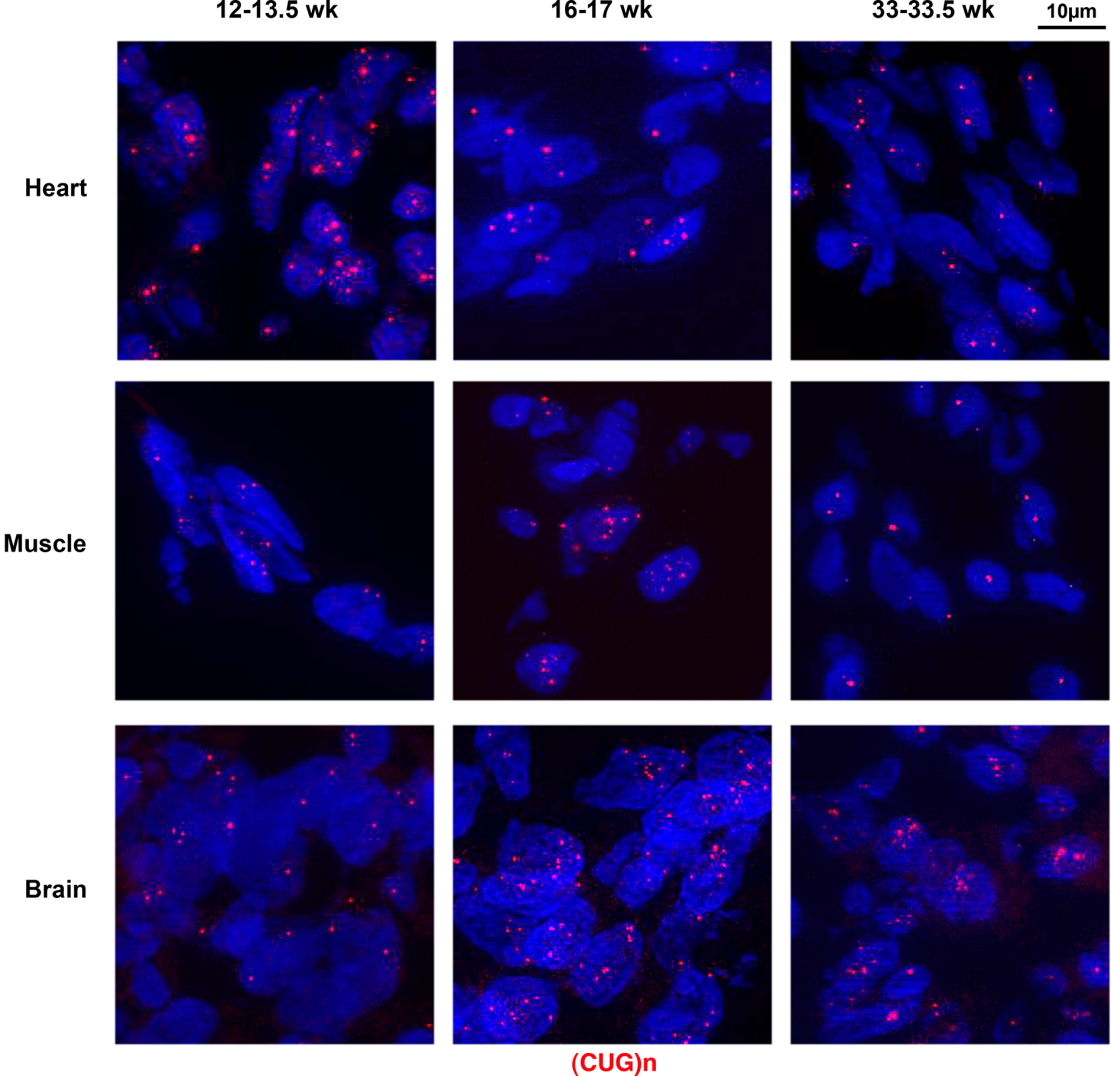
Mutant sense *DMPK* RNA nuclear foci in human DM1 fetal samples. RNA foci containing (CUG)n expansion were labeled in red, using a 5’-Cy3-labeled (CAG)_5_ PNA probe in heart, skeletal muscle and brain samples from 12–13.5 week-old (12–13.5 wk), 16–17 week-old (16–17 wk) and 33–33.5 week-old (33–33.5 wk) DM1 fetuses.

Using (CAG)_5_ fluorescent probe, we investigated if we could detect expanded *DMPK* sense RNA foci, one of the hallmarks of DM1, in human fetal tissues samples. Interestingly, we observed very abundant foci in heart, skeletal muscle and brain as early as 12 weeks of gestation. RNA foci were also detected in these 3 tissues at later stages, up to 33.5 weeks (Fig 1), despite the fact that samples were kept at-80° for at least 18 years. In order to get an estimation of the percentage of nuclei showing foci in the different tissues and at various fetal ages, we quantified the number of foci observed per nucleus using a 3D software. All the samples showed that at least 69% up to 97% of the observed nuclei contained expanded *DMPK* sense RNA foci. We studied the distribution of foci per nucleus (S1 Fig), and found that in some cases, a high number of nuclear aggregates was detected in a single nucleus (>10 foci per nucleus, S1 Fig). However, This quantification cannot be used to precisely compare the foci distribution between different tissues as the samples were very old and their integrity variable from one sample to another.

**Fig 2:**
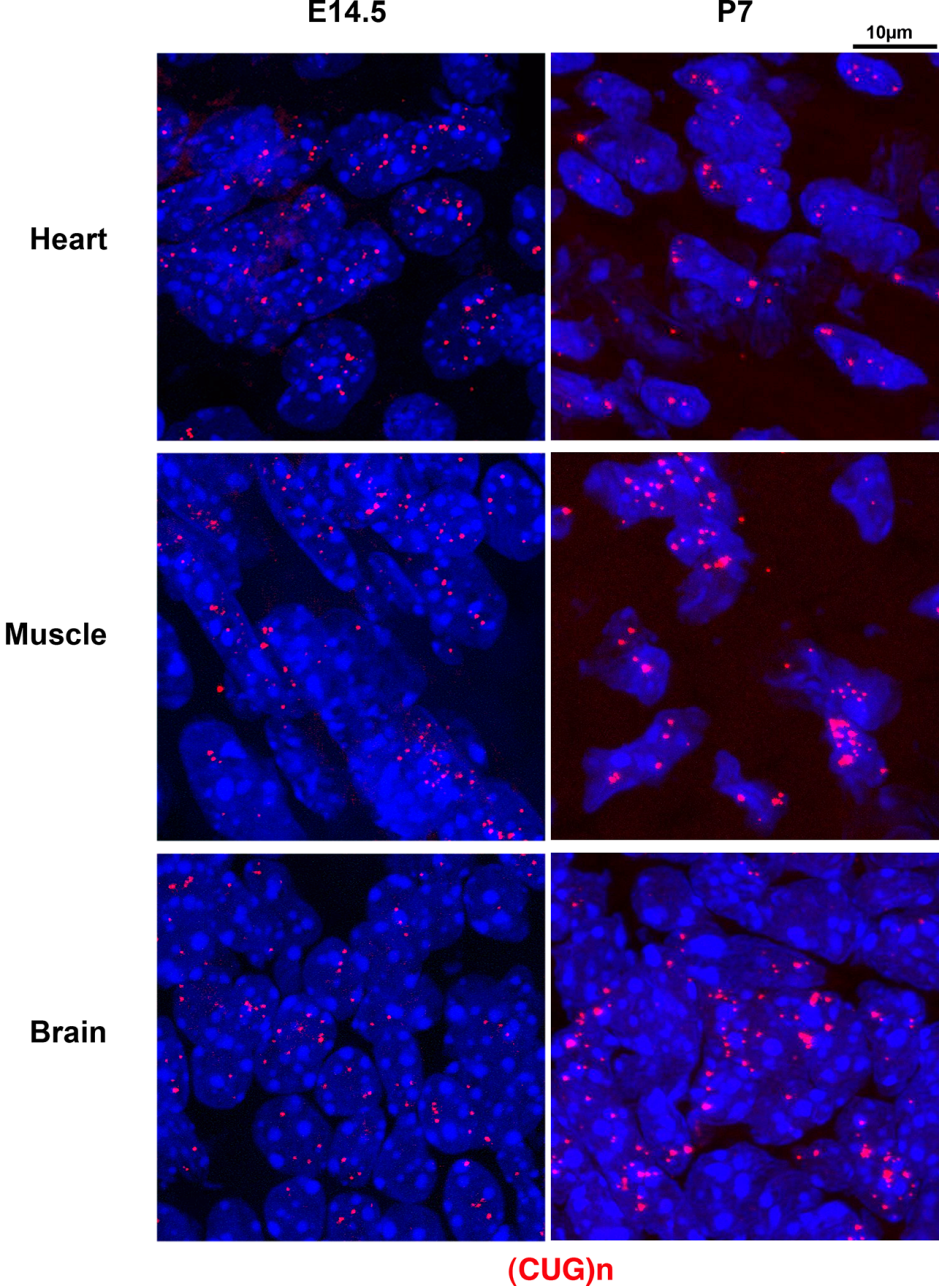
Mutant sense *DMPK* RNA nuclear foci in DMSXL embryos and neonates. RNA foci containing (CUG)n expansion were labeled in red, using a 5’-Cy3-labeled (CAG)_5_ PNA probe in E14.5 embryos heart, muscle (hind leg) and brain (cortex) and P7 neonates heart (ventricule), muscle (gastrocnemius) and brain (frontal cortex).

**Fig 3:**
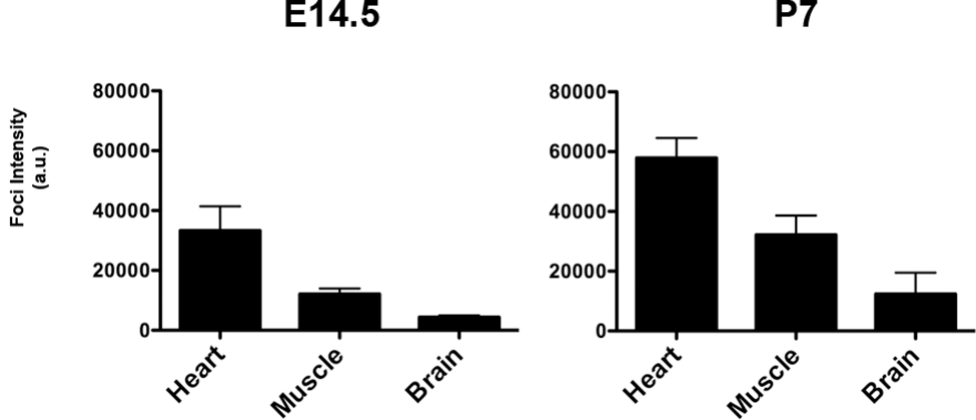
Quantification of mutant sense *DMPK* RNA nuclear foci intensity in DMSXL embryos and neonates. Intensity was measured in three E14.5 embryos heart, muscle (hind leg) and brain (cortex) and three P7 neonates heart (ventricule), muscle (gastrocnemius) and brain (frontal cortex). The graph represents the mean of the normalized intensity (total intensity/number of nuclei in arbitrary units, a.u.) ± SEM.

We investigated the presence of expanded *DMPK* sense RNA foci at early stages in the DMSXL mouse model. DMSXL are transgenic mice carrying the human *DMPK* gene with >1000 CTG that is expressed in various tissues under the control of its own human promoter and regulatory sequences [32]. Using *in situ* hybridization, we observed a very high number of mutant RNA foci in heart, skeletal muscle and brain from DMSXL embryos at embryonic day 14.5 (E14.5) and DMSXL neonates on postnatal day 7 (P7) (Fig 2). At both E14.5 and P7 stages, foci were very abundant in all cells, with a high number of foci per nucleus. Quantification of foci intensity, using confocal microscopy and normalization for the number of nuclei analyzed, demonstrated higher intensity in heart, followed by muscle and then brain at both E14.5 and P7 stages (Fig 3).

**Fig 4:**
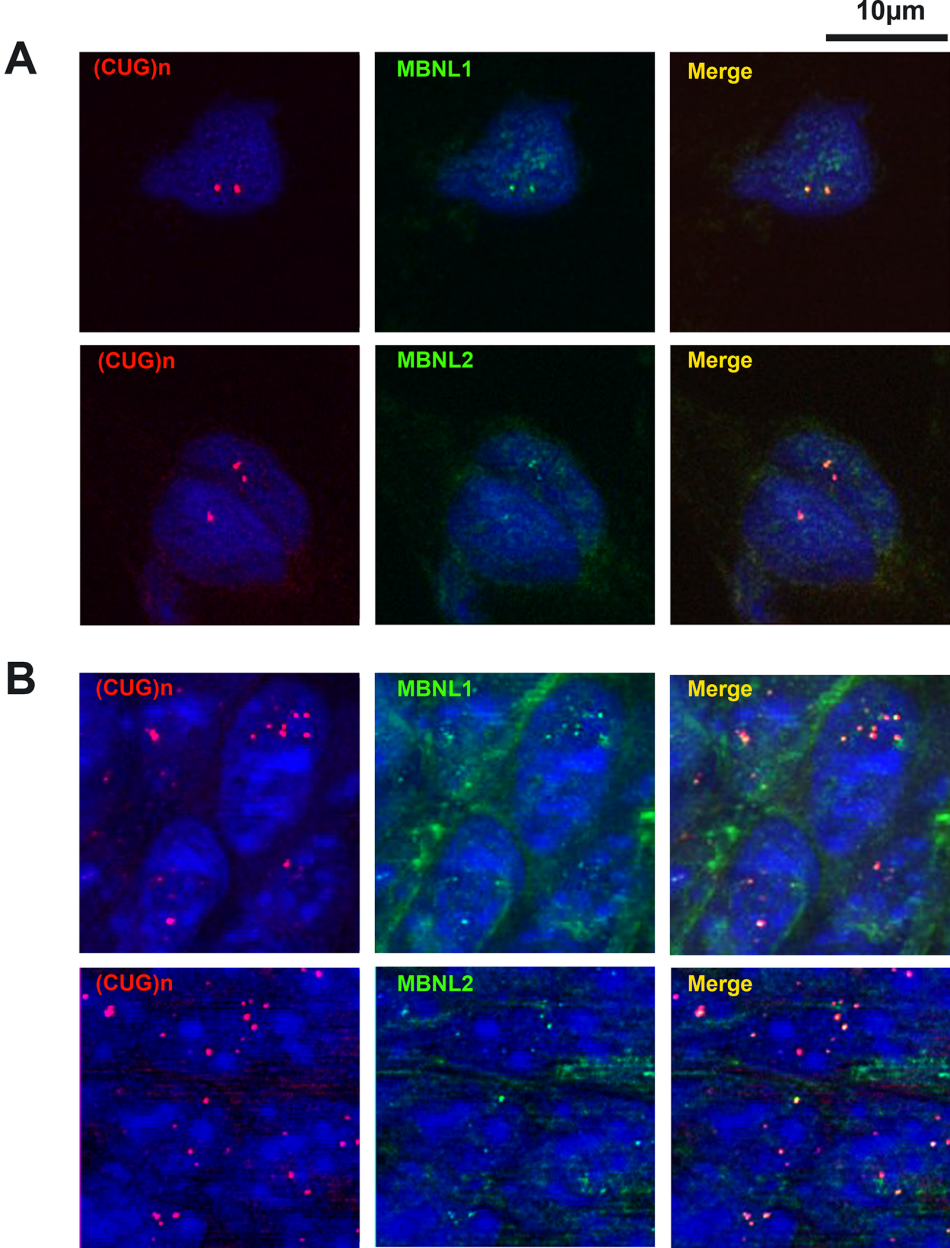
Co-localization of the mutant sense *DMPK* RNA nuclear foci with MBNL1 and MBNL2 proteins. *In situ* hybridization was combined with immunofluorescence in skeletal muscle tissues from a human DM1 13.5 week-old fetus (A) and from a DMSXL E14.5 muscle hind leg (B). Left panels show hybridization with a red fluorescent probe recognizing expanded (CUG)n sense *DMPK* RNA transcripts, middle panels show immunofluorescence using MBNL1 or MBNL2 antibodies and right panels merged images showing RNA and protein co-localization.

In DM1 adult form, it has been shown that mutant RNA foci sequester MBNL proteins. As MBNL proteins are developmentally regulated [33–35], we investigated if mutant *DMPK* sense RNA foci sequestered MBNL1 and MBNL2 in embryonic and fetal DM1 tissues. Using combined *in situ* hybridization and immunohistochemistry, we observed that the majority of RNA foci co-localized with MBNL1 and MBNL2 in heart, muscle and brain from human DM1 fetal samples and from DMSXL embryonic and postnatal samples (Fig 4 and data not shown).

### Expanded *DMPK* antisense transcripts nuclear foci are also detected during development

We previously showed that the antisense transcripts detected in 3’UTR of the *DMPK* gene encompassed the expanded CAG repeat and were able to form antisense RNA nuclear foci in adult DM1 and DMSXL adult tissues [32]. Thus, we investigated the accumulation of this antisense RNA in DM1 affected tissues (heart, skeletal muscle and brain) during development.

**Fig 5:**
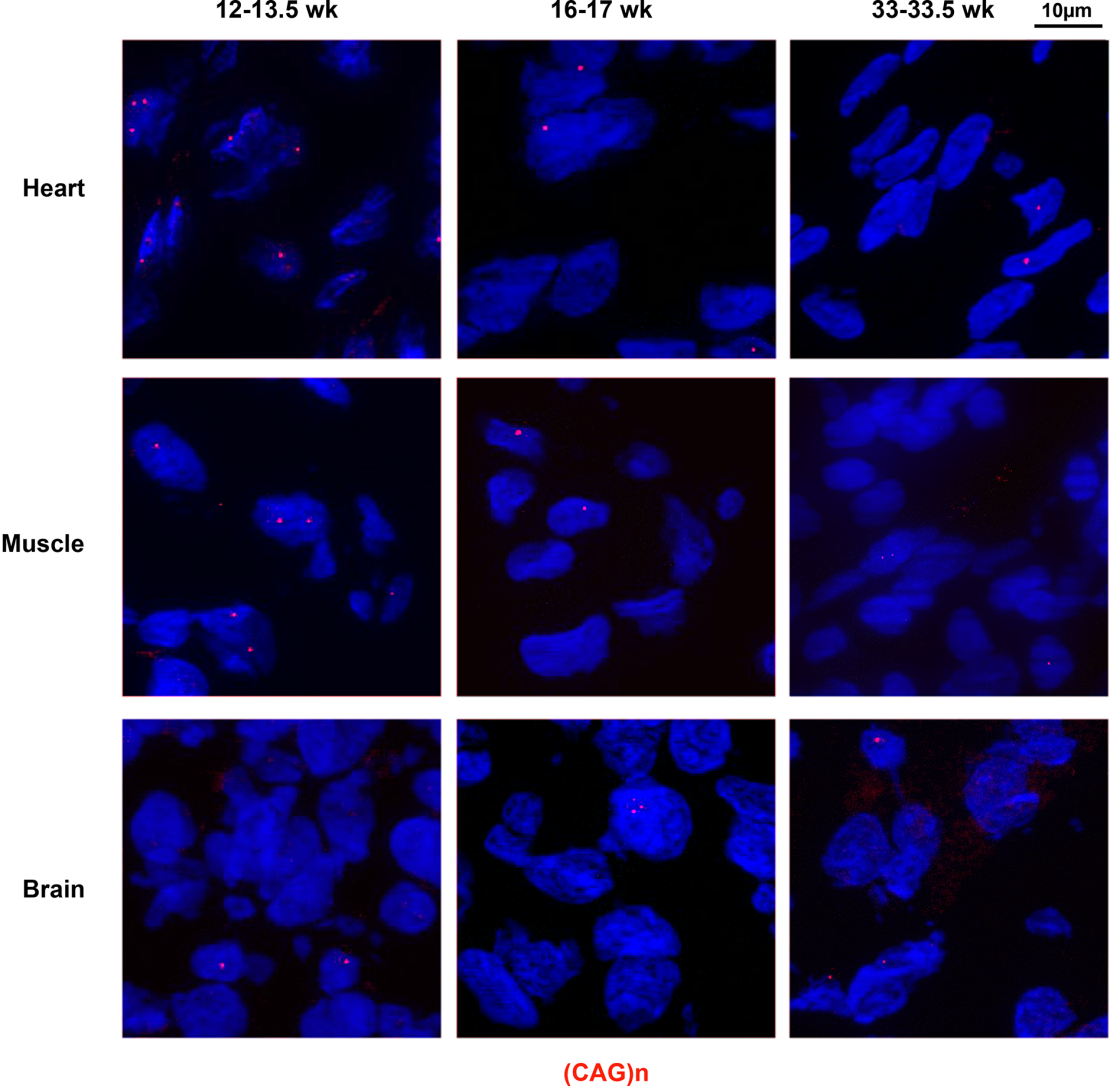
Mutant antisense *DMPK* RNA nuclear foci in human DM1 fetal samples. RNA foci containing (CAG)n expansions were labeled in red, using a 5’-Cy3-labeled (CTG)_5_ PNA probe in heart, skeletal muscle and brain samples from 12–13.5 week-old (12–13.5 wk), 16–17 week-old (16–17 wk) and 33–33.5 week-old (33–33.5 wk) DM1 fetuses.

Using a (CTG)_5_ fluorescent probe, we observed mutant antisense *DMPK* RNA foci in heart, skeletal muscle and brain from 12 to 33.5 week old human fetuses (Fig 5). However, antisense *DMPK* foci were less numerous than sense *DMPK* foci and appeared fainter.

**Fig 6:**
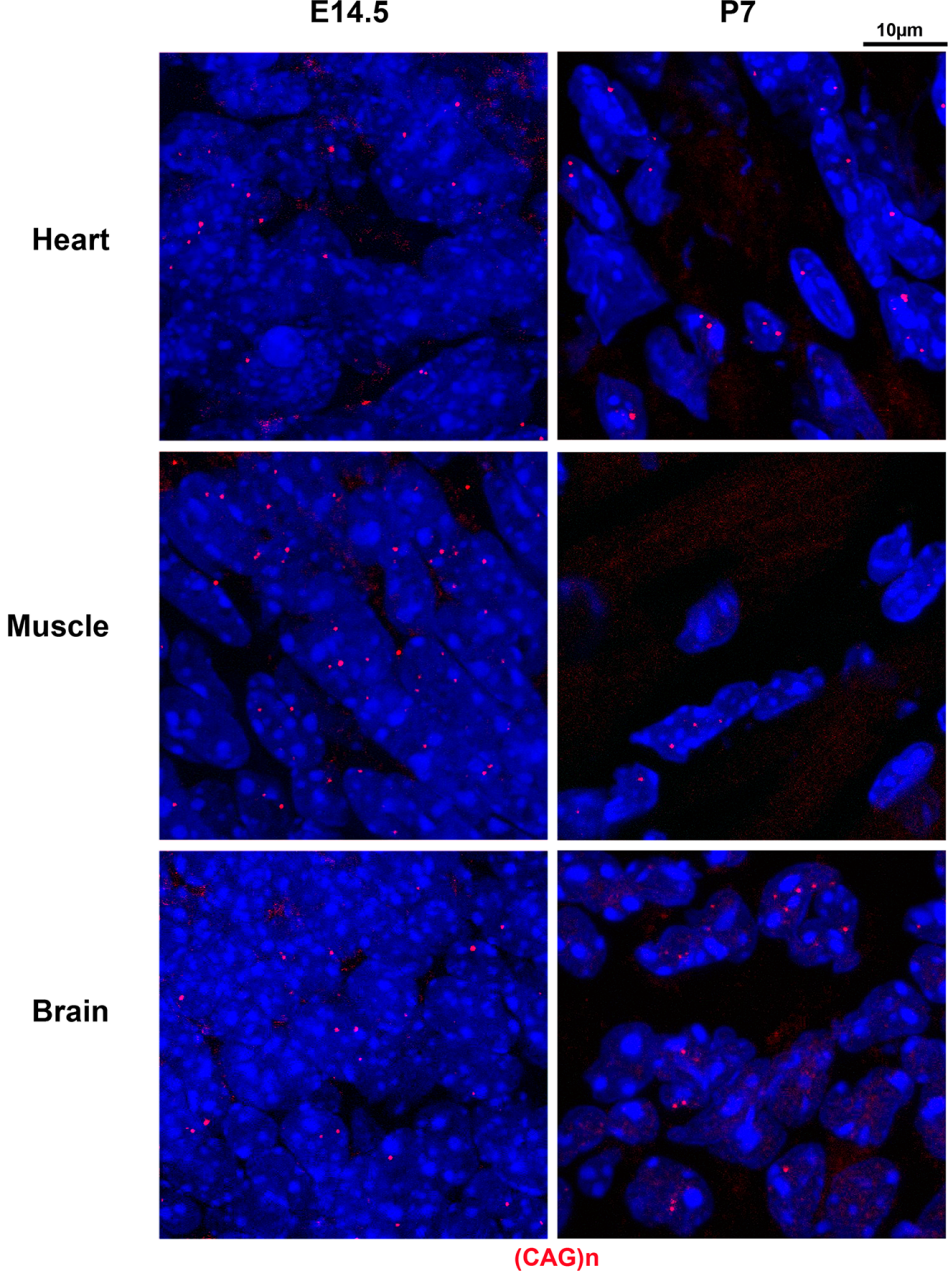
Mutant antisense *DMPK* RNA nuclear foci in DMSXL embryos and neonates. RNA foci containing (CAG)n expansions were labeled in red, using a 5’-Cy3-labeled (CTG)_5_ PNA probe in E14.5 embryos heart, muscle (hind leg) and brain (cortex) and P7 neonates heart (ventricule), muscle (gastrocnemius) and brain (frontal cortex).

**Fig 7:**
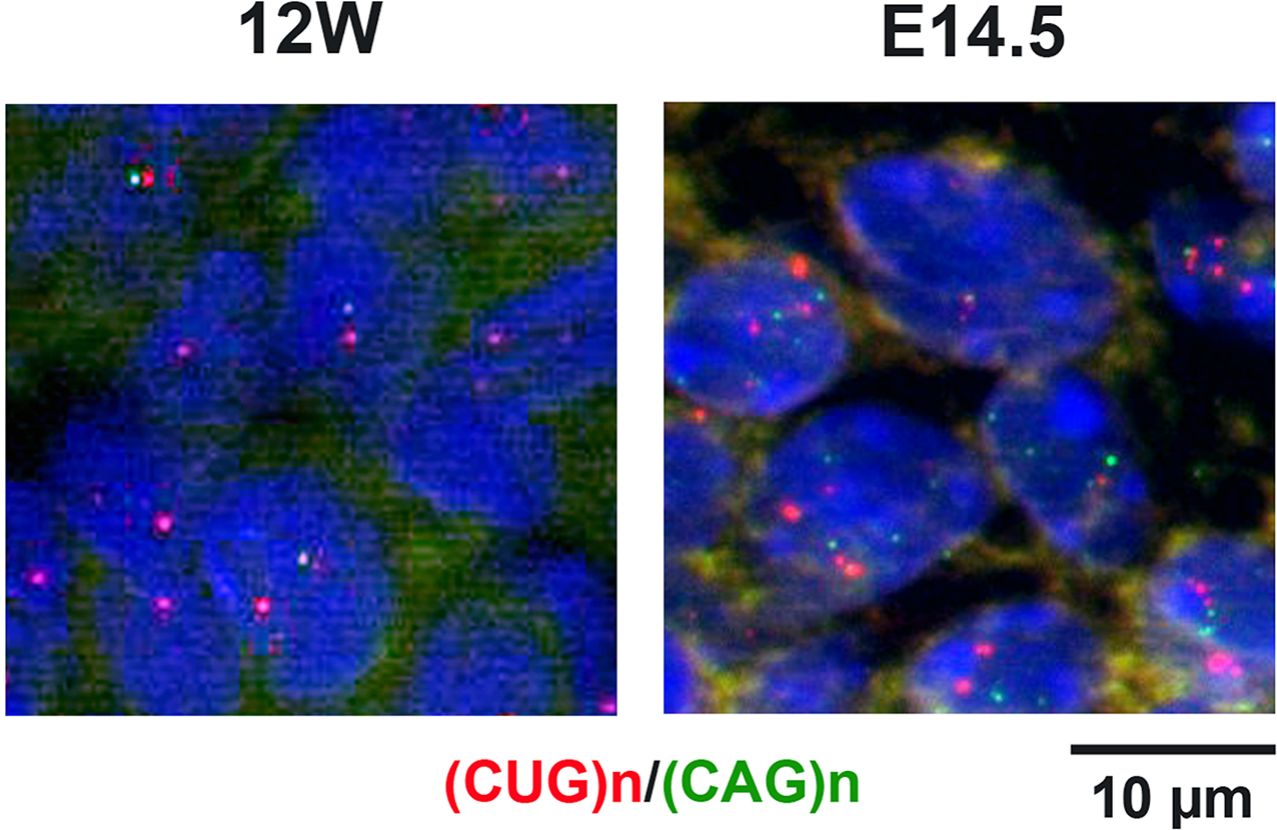
Simultaneous detection of mutant sense and antisense *DMPK* RNA nuclear foci. Localization of sense and antisense *DMPK* foci was studied using 5’-cy3-(CAG)5 (recognizing sense (CUG)n transcripts in red) and 5’-Alexa 488-(CTG)5 (recognizing antisense (CAG)n transcripts in green) probes in the same experiment. Left panel: human 12 week-old fetus heart sample; right panel: DMSXL E14.5 embryonic muscle from the hind leg.

In DMSXL embryos and neonates, we also detected mutant antisense *DMPK* RNA foci (Fig 6). In both human and mouse samples we verified that the antisense foci disappeared with RNase treatment but not with DNase treatment (S2 Fig). The antisense RNA foci were observed in heart, skeletal muscle and brain at E14.5 and P7 in DMSXL (Fig 5), and appeared quite abundant in E14.5 embryos (although they remained less abundant than sense foci, compare Figs 2 and 6). Fig 7 shows that sense and antisense *DMPK* RNA foci, although present in the same nucleus, do not co-localize. We explored the possible co-localization of the antisense foci with MBNL1 or MBNL2. This was technically difficult, given the high background of the (CTG)n probe combined with MBNL antibodies, resulting in the detection of very faint foci. Nevertheless, in those cells where we could observe antisense foci, they did not appear to co-localize with MBNL proteins (data not shown).

### Sense and antisense *DMPK* transcripts are expressed during development

**Fig 8:**
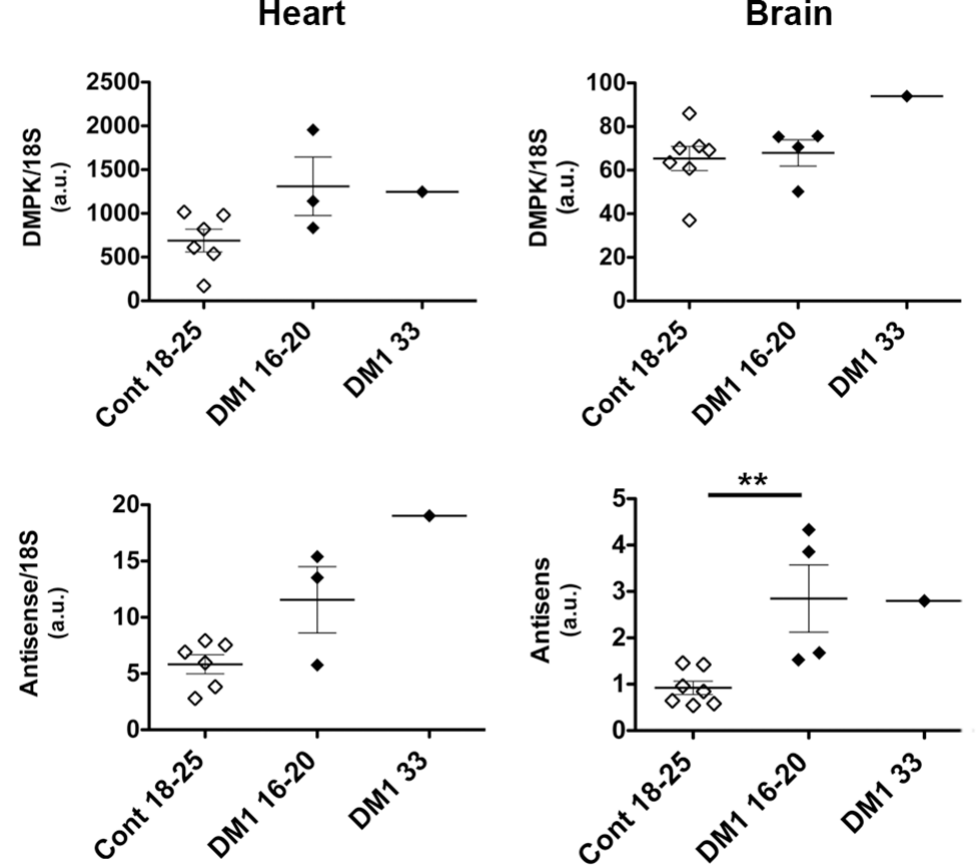
Sense and antisense *DMPK* RNA levels in DM1 fetuses. qRT-PCR were performed in heart (left panels) and in brain (right panels) using specific primers and standard curves established with a known number of plasmid molecules with the amplicons as described previously [32]. Levels of sense and antisense *DMPK* transcripts were reported on graphs using 18S as internal control, in arbitrary units (a.u.). Cont 18–25: samples from control fetuses aged between 18–25 weeks. DM1 16–20: samples from DM1 fetuses aged between 16–20 weeks. DM1 33: sample from a 33 weeks old DM1 fetus. The mean and SEM are represented respectively, as horizontal and vertical bars. (**, p<0.01, two-tailed Student’s t-test).

Using specific qRT-PCR and standard curves established with a known number of plasmid molecules coding the amplicons, we studied the levels of sense and antisense *DMPK* transcripts in control and DM1 fetal heart and brain. Unfortunately, we did not have enough tissues to extract RNA from fetal muscle samples. Fig 8 shows that sense *DMPK* transcripts are detected at higher levels in heart than in brain samples. No statistical significant difference was observed between control and DM1 samples of the same ages. Antisense *DMPK* transcripts are expressed at lower level when compared to sense transcripts both in heart and brain. Interestingly, levels of antisense transcripts appeared increased in DM1 heart and brain compared to control samples. The difference reached significance only in brain (we considered only the 4 age-matched samples aged between 16–20 weeks, p = 0.0061).

**Fig 9:**
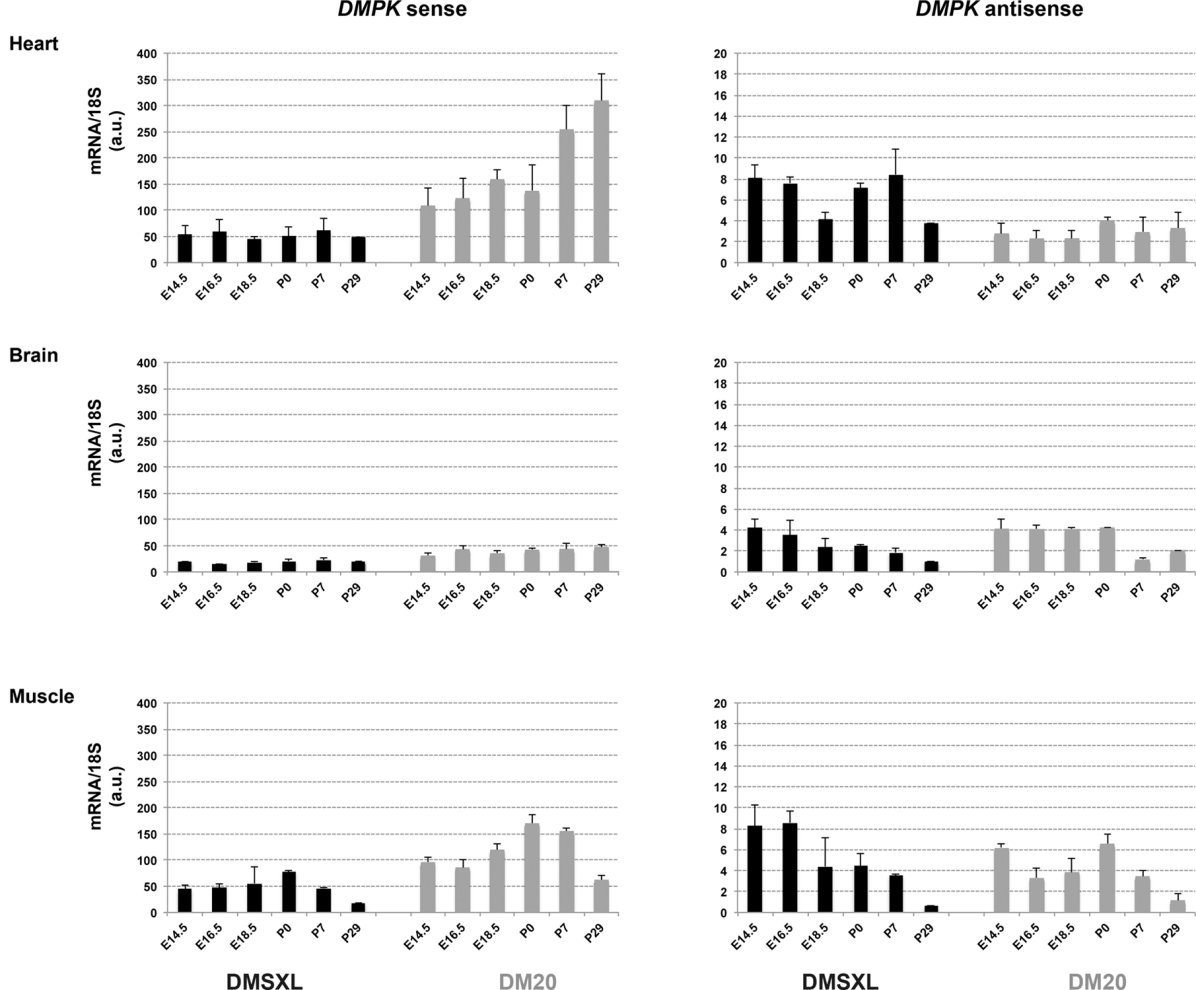
Sense and antisense *DMPK* RNA levels in transgenic mice. qRT-PCR were performed in heart, skeletal muscle and brain from DMSXL and DM20 embryos and neonates at embryonic E14.5 to postnatal P29 stages. Levels of sense *DMPK* (left panels) and antisense transcripts (right panels) were reported on graphs using 18S as internal control, in arbitrary units (a.u.) with standard deviation of the mean for repeated experiments.

In order to get more information on the developmental regulation of *DMPK* sense and antisense RNA over a wider development period, we studied by qRT-PCR the levels of transcripts in transgenic mice from E14.5 up to postnatal age 29 days (P29) (Fig 9). We compared mice carrying one copy of the transgene with >1000 CTG (DMSXL) and mice carrying one copy of the transgene with a normal 20 CTG repeat (DM20). As in adult DMSXL mice and human [32], levels of sense *DMPK* RNA were higher in heart and in muscle compared to levels observed in brain in both lines. In the DM20 line, sense *DMPK* transcripts increased during development, similar to the endogenous sense *Dmpk* transcripts, except at P29 in muscle (S3 Fig). In DMSXL, levels of sense transcripts are lower than in DM20 and the increase during development is less obvious especially during heart development. Interestingly, the levels of antisense transcripts in DMSXL and DM20 are similar in muscle and brain and are even higher in DMSXL heart compared to DM20 heart (Fig 9). Furthermore, in both lines the developmental expression pattern was different for sense and antisense transcripts.

## Discussion

During the last decade, numerous studies have clearly demonstrated that expanded *DMPK* RNA are the central player in DM pathogenesis and exhibit a deleterious gain-of-function. The molecular hallmark of *DMPK* RNA toxicity in patients is the formation of abnormal nuclear foci formed by aggregation of the expanded transcripts [10]. These foci then disturb the function of nuclear proteins (including MBNL and CELF proteins) involved in various regulatory processes that are in turn affected [12]. If the toxic effects of expanded *DMPK* transcripts have been well documented in the case of adult DM1, less is known about the formation of toxic RNA foci during development in DM1 and CDM fetal tissues.

In order to gain insight into the mechanisms underlying CDM that affect babies at birth, we studied the formation of sense and antisense *DMPK* RNA foci, during development in DM1 fetal tissues and in DMSXL mice, in the major affected tissues: heart, skeletal muscle and brain. In all three tissues, we observed very abundant sense *DMPK* RNA nuclear foci as early as 12 weeks in DM1 fetuses and E14.5 in DMSXL mouse embryos. In mouse embryos and neonates, the highest intensity was observed in heart. The foci intensity observed are consistent with the profile of *DMPK* expression that is higher in heart and muscle at both stages (Fig 9).

Furthermore, we found that sense *DMPK* foci co-localized with MBNL1 and MBNL2. These results show that the mutant sense *DMPK* RNA is potentially toxic from very early stages and that the mechanisms underlying CDM operate early on during development, although no major morphological developmental features are observed in CDM neonates. Immaturity and high mortality observed for CDM patients, as well as for DMSXL young mice [32], certainly involved early toxicity of mutant *DMPK* RNA. We also found that the mutant antisense *DMPK* RNA, more recently identified [36], can also form RNA nuclear foci like expanded sense *DMPK* transcripts at the same developmental stages. However, antisense RNA foci are much less abundant. Furthermore, we could not detect their co-localization with MBNL proteins. We observed that the antisense foci do not co-localize with the sense foci, even though they can accumulate in the same nucleus. This indicates that both sense and antisense transcripts can be expressed in the same cell and can form foci independently. The role of antisense expanded transcripts in DM1 is still unknown. Even if MBNL proteins can bind CAG repeats [37], it is unlikely that antisense foci sequester a sufficient amount of MBNL proteins to be toxic. However, it has been clearly demonstrated that CAG expanded repeats can generated potentially toxic polypeptides via RAN translation [38]. These polypeptides could also play a role in CDM.

In order to better characterize the expression of sense and antisense *DMPK* RNA with normal or expanded repeats, we measured the levels of expression in control and affected DM1 fetuses as well as in transgenic embryos and neonates carrying 20 or >1000 CTG repeats. No significant difference was observed for sense *DMPK* expression between control or affected samples. In contrast, antisense *DMPK* transcripts showed a significant increase in affected brain samples and a tendency for augmentation in affected DM1 heart. This observation suggests that expression of the antisense transcripts, for which the regulatory sequences are located at about 1 kb downstream of the CTG repeat, is deregulated by the CTG expansion while the sense transcripts is less affected. These results must be considered cautiously as they were obtained with a limited number of old human samples with variable integrity. To get a better view of sense and antisense *DMPK* regulation through embryonic and fetal development, we used the DM20 and DMSXL transgenic mice. We previously showed that the pattern of *DMPK* expression in tissues is similar to the pattern observed for the human *DMPK* gene in human samples, suggesting that the majority of the regulatory sequences are comprised in the transgenes [30, 32]. Thus we investigated how sense and antisense *DMPK* RNA are regulated during development from embryonic E14.5 to neonatal ages. Interestingly, Fig 9 showed that the regulation of the sense and antisense transcripts are different for both lines. In DM20 mice, sense *DMPK* RNA increase during development similar to the mouse *Dmpk* gene, while this is not the case for antisense *DMPK* RNA. Together, these profiles suggest that the regulation of the two transcripts is independent in the presence of short repeats. They do not appear to be regulated in mirror, suggesting that there are not regulating each other, as it has been described for others bidirectional transcripts [39, 40]. In contrast with DM20 or the endogenous *Dmpk* transcripts, in DMSXL sense *DMPK* transcripts do not clearly increase during heart development. It has been proposed that expanded repeats could affect *DMPK* expression by inducing hypermethylation around the repeat, disturbing the binding of CTCF and subsequently the expression of *DMPK* [23, 41]. In line with this hypothesis, the expression of sense *DMPK* RNA is reduced in DMSXL compared to DM20 heart, muscle and brain. However, the expression of antisense transcript is similar in muscle and brain and increased in DMSXL heart. The difference observed between DMSXL and DM20 may be explained by the disturbance of sense and antisense *DMPK* regulation due to the expanded repeat. However, it has to be noted that the transgene (one copy) is integrated in different locations in DMSXL and DM20 mice and we cannot exclude a position effect on the regulation of *DMPK*. Furthermore, the developmental pattern of sense *DMPK* expression is different in both transgenic mice (DMSXL and DM20) compared to the endogenous *Dmpk* gene in muscle with a decrease of *DMPK* sense transcript in mice at P29. This could reflect also position effect or inadequate regulation of the human *DMPK* expression (with normal or expanded repeat) by mouse transcription factors in muscle at later stage.

In conclusion, we showed that sense and antisense *DMPK* transcripts are expressed from early stages during development suggesting that expanded RNA could trigger pathogenic events at early stages, contributing to developmental disease features, such as mental retardation and muscle immaturity described in CDM. Sense foci clearly sequester MBNL protein at early stages demonstrated that MBNL proteins as in adult DM1 are probably also a pathological intermediate in CDM. Antisense transcripts that appeared to be expressed in affected tissues and for which expression is higher at early stages could also participate to RNA toxicity but independently of MBNL proteins that do not appeared sequestered at high levels by the antisense foci. RAN translation is an appealing possible mechanism of toxicity and could have more impact at early stages when the antisense transcripts are expressed at higher levels.

## Supporting Information

S1 Fig Distribution and frequency of the mutant *DMPK* RNA nuclear foci per nucleus in human DM1 fetal samples. The number of foci per nucleus was determined using 3D images. Graphs represent the percentage of cells showing a given number of foci per nucleus. A: heart; B: skeletal muscle; C: brain, 12–13 wk: 12 to 13 week-old fetuses; 16–17 wk: 16 to 17 week-old fetuses; 33–33.5 wk: 33 to 33.5 week-old fetuses.
(TIF)

S2 Fig Mutant antisense *DMPK* RNA nuclear foci in human DM fetal and DMSXL neonate samples disappear after RNase treatment but not after DNase treatment. RNA foci containing (CAG)n expansions were labeled in red, using a 5’-Cy3-labeled (CTG)_5_ PNA probe in heart samples from a 12 week-old DM fetus (DM-12wk), and in DMSXL P7 neonates heart samples (DMSXL-P7), with or without treatment with RNase (+ RNase) or DNase (+DNase).
(TIF)

S3 Fig Sense mouse *Dmpk* RNA levels in mice. Mouse endogenous sense *Dmpk* transcripts were studied by qRT-PCR in heart, skeletal muscle and brain from DMSXL embryos and neonates at embryonic E14.5 to postnatal P29 stages. Levels of *Dmpk* transcripts were reported on graphs using 18S as internal control, in arbitrary units (a.u.) with standard deviation of the mean for repeated experiments.
(TIF)
