# Supplementary figures and images for: Sense and Antisense DMPK RNA Foci Accumulate in DM1 Tissues during Development
Source: PLoS One. 2015 Sep 4;10(9):e0137620. doi: 10.1371/journal.pone.0137620 (PMC4560382; doi:10.1371/journal.pone.0137620)

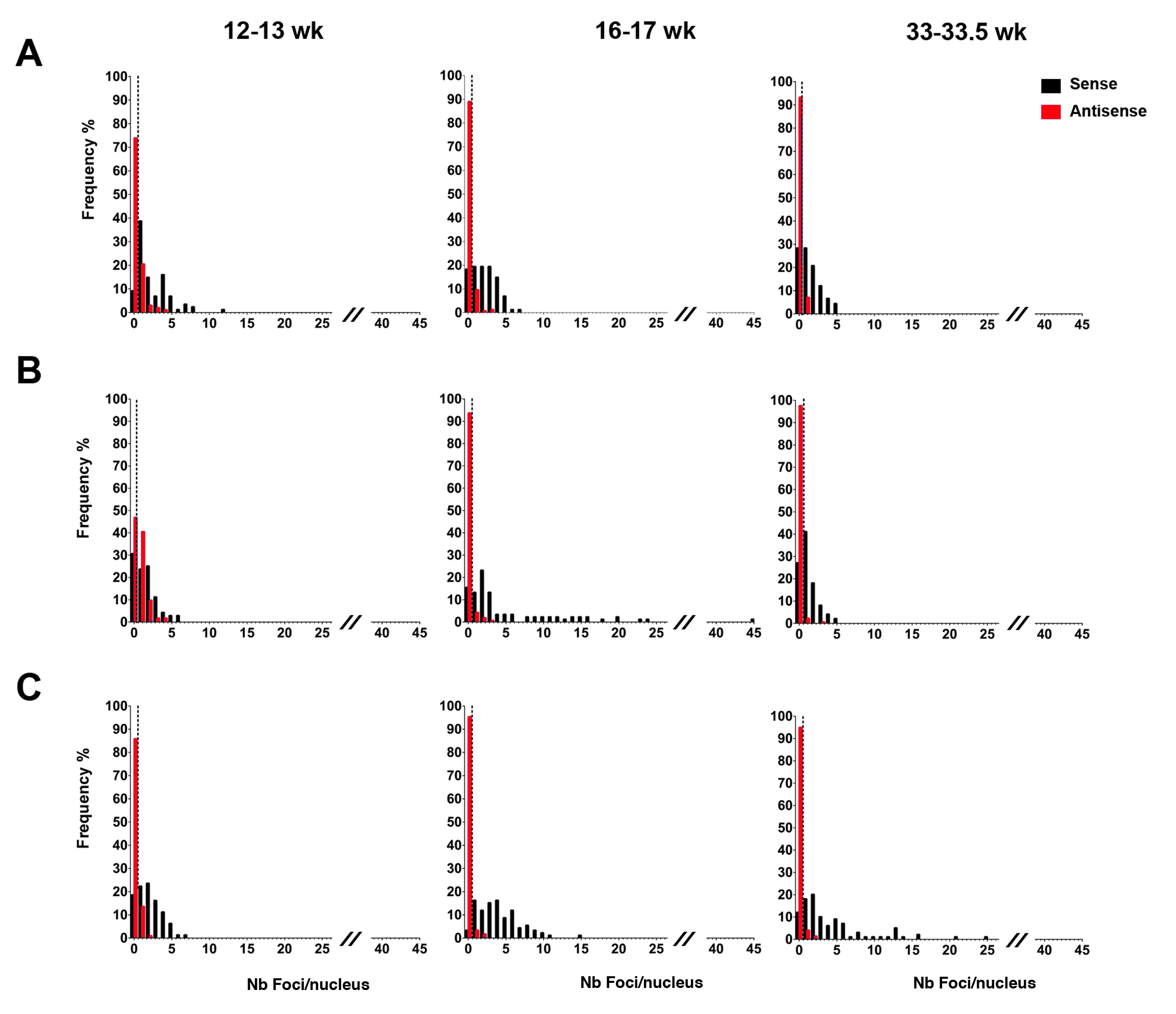

Supplement: S1 Fig — The number of foci per nucleus was determined using 3D images. Graphs represent the percentage of cells showing a given number of foci per nucleus. A: heart; B: skeletal muscle; C: brain, 12–13 wk: 12 to 13 week-old fetuses; 16–17 wk: 16 to 17 week-old fetuses; 33–33.5 wk: 33 to 33.5 week-old fetuses. (TIF) [file pone.0137620.s001.tif]

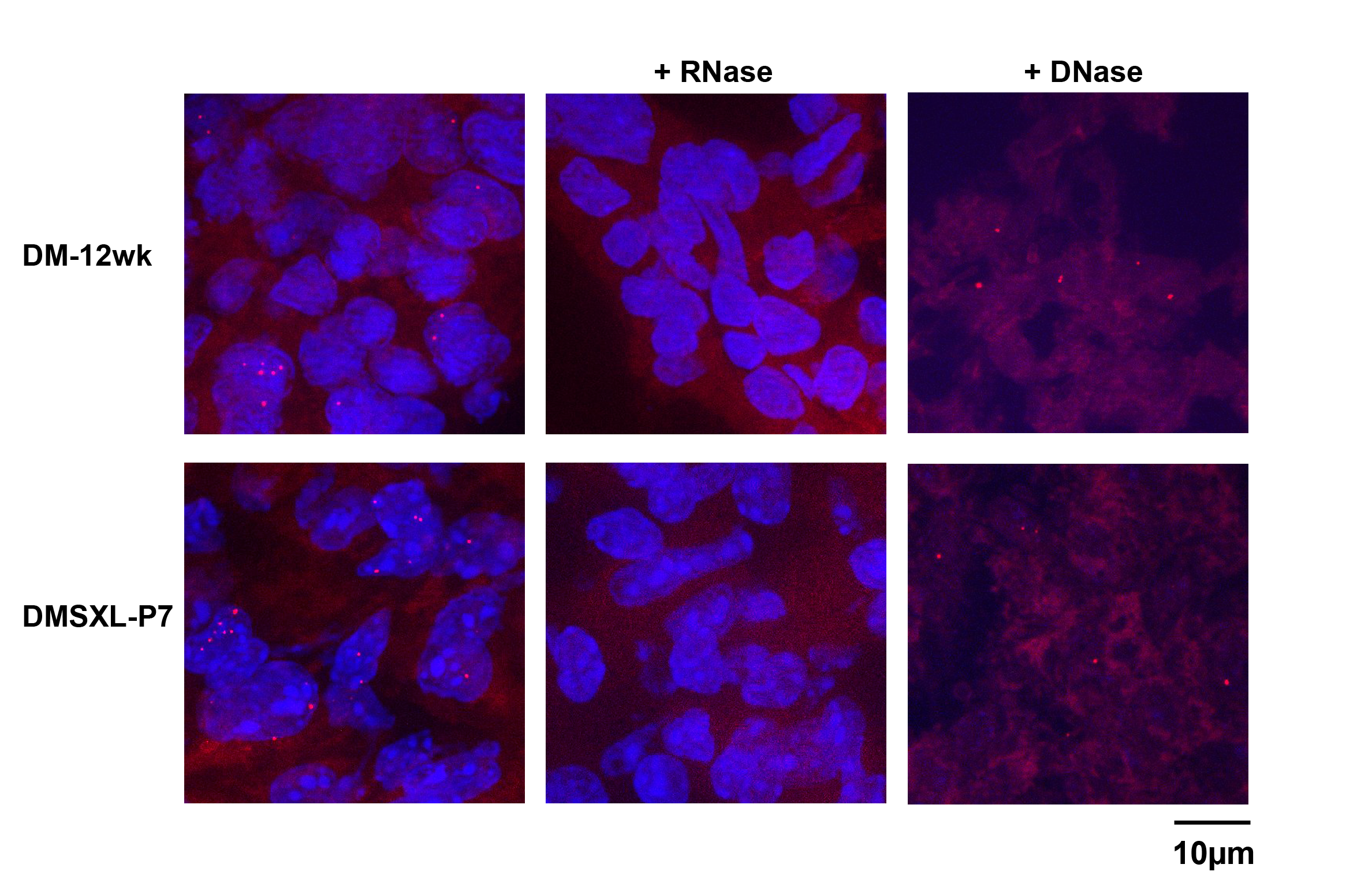

Supplement: S2 Fig — RNA foci containing (CAG)n expansions were labeled in red, using a 5’-Cy3-labeled (CTG)5 PNA probe in heart samples from a 12 week-old DM fetus (DM-12wk), and in DMSXL P7 neonates heart samples (DMSXL-P7), with or without treatment with RNase (+ RNase) or DNase (+DNase). (TIF) [file pone.0137620.s002.tif]

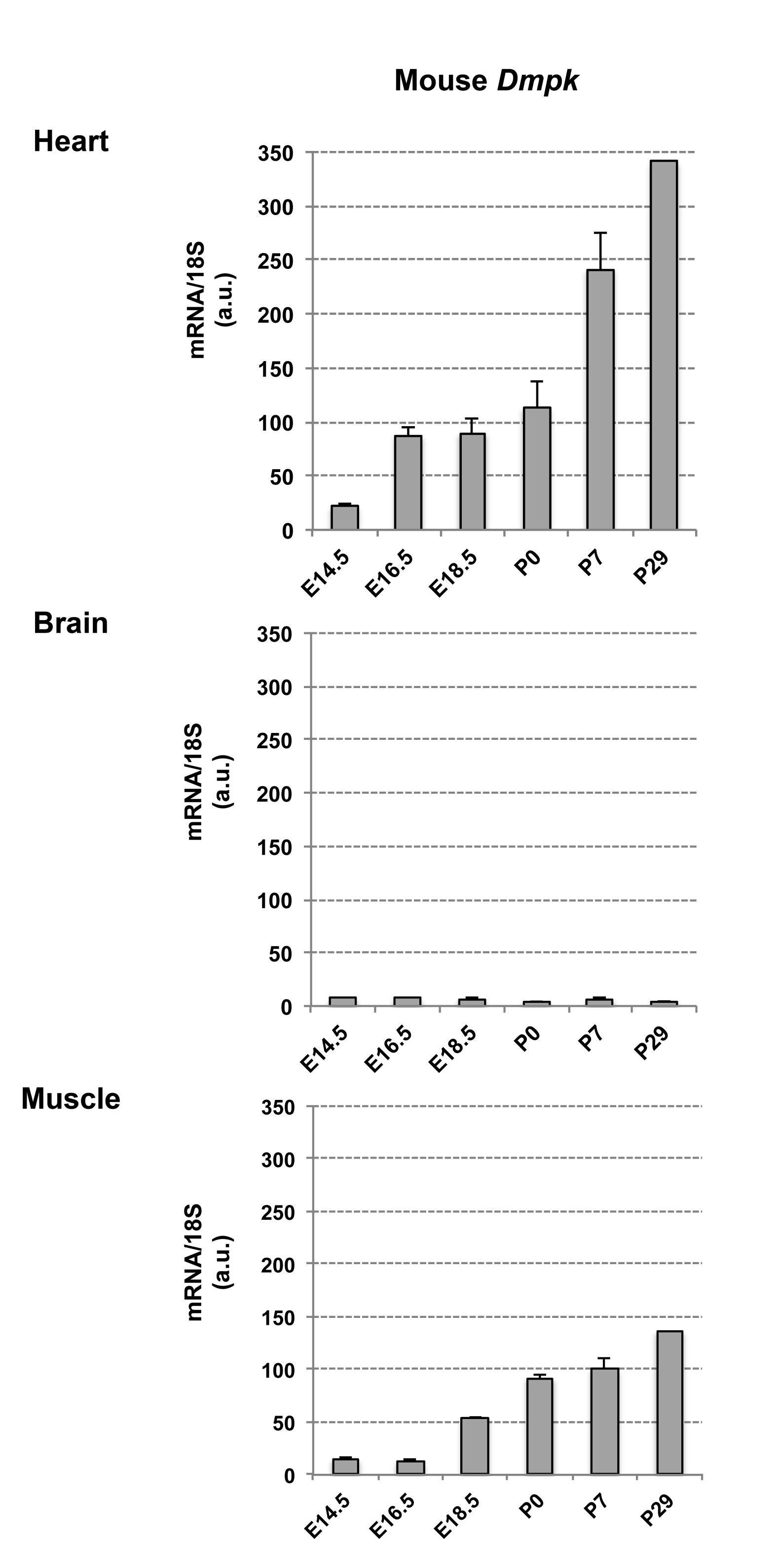

Supplement: S3 Fig — Mouse endogenous sense Dmpk transcripts were studied by qRT-PCR in heart, skeletal muscle and brain from DMSXL embryos and neonates at embryonic E14.5 to postnatal P29 stages. Levels of Dmpk transcripts were reported on graphs using 18S as internal control, in arbitrary units (a.u.) with standard deviation of the mean for repeated experiments. (TIF) [file pone.0137620.s003.tif]
